# Supplementary material for: Identification of photoperiod-induced specific miRNAs in the adrenal glands of Sunite sheep (Ovis aries)
Source: Front Vet Sci. 2022 Jul 22;9:888207. doi: 10.3389/fvets.2022.888207 (PMC9354845; doi:10.3389/fvets.2022.888207)
Supplement: Supplementary file 1 [file Data_Sheet_1.ZIP › Supplementary file/miRDeep2 file.zip!/miRDeep2 file/Novel_Structure/SPLP42A2/Novel_700.pdf]

Provisional ID	:	NC_019483.2_41959
Score total	:	2.6
Score for star read(s)	:	-1.3
Score for read counts	:	0
Score for mfe	:	2.3
Score for randfold	:	1.6
Score for cons. seed	:	
Total read count	:	29
Mature read count	:	29
Loop read count	:	0
Star read count	:	0

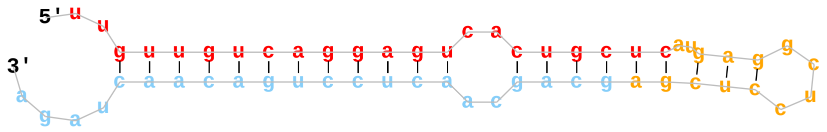

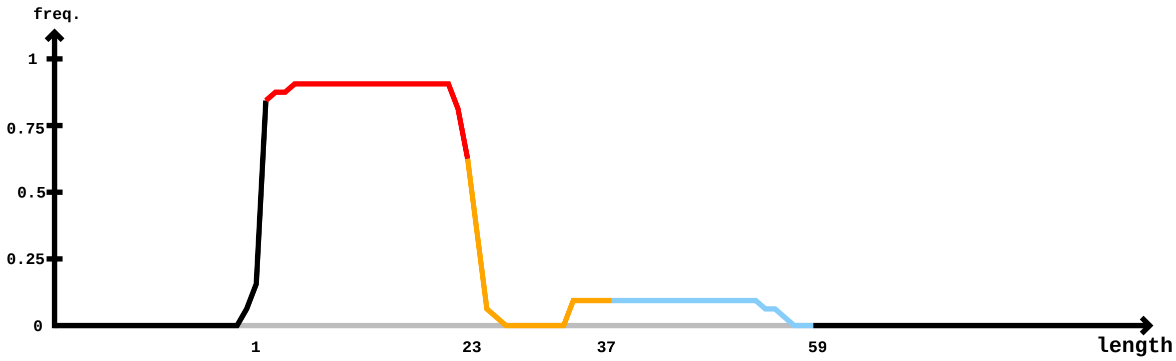

Mature Star

```
5'- gacgauguaggcucacccacuuguugucaggagucacugcucaugaggcuccucgagcagcaacuccugacaacuagaagggagggcagagcaucagcgcagccccugugga  -3'    exp
    ...(((((..((((.(((.((.((((((((((((..((((((..(((....)))))))))..)))))))))))).))..))).))))...)))))..(((((...)))))..   reads    mm    sample
    ..................acuuguugucaggagucacugc........................................................................   2        0     seq
    ...................cuuguugucaggagucacugcu.......................................................................   1        0     seq
    ...................cuuguugucaggagucacugcuc......................................................................   1        0     seq
    ...................cuuguugucaggagucacugcuca.....................................................................   1        0     seq
    ....................uuguugucaggagucacugc........................................................................   1        0     seq
    ....................uuguugucaggagucacugcu.......................................................................   4        0     seq
    ....................uuguugucaggagucacugcuc......................................................................   11       0     seq
    ....................uuguugucaggagucacugcuca.....................................................................   5        0     seq
    ....................uuguugucaggagucacugcucau....................................................................   1        0     seq
    .....................uguugucaggagucacugcu.......................................................................   1        0     seq
    .......................uugucaggagucacugcucaug...................................................................   1        0     seq
    ....................................................ucgagcagcaacuccugaca........................................   1        0     seq
    ....................................................ucgagcagcaacuccugacaac......................................   1        0     seq
    ....................................................ucgagcagcaacuccugacaacu.....................................   1        0     seq
```
